# Supplementary material for: Effect of SARS-CoV-2 infection in neonates or in pregnancy on developmental outcomes at 21–24 months (SINEPOST): study protocol for a prospective cohort study
Source: BMJ Paediatr Open. 2022 Sep 19;6(1):e001571. doi: 10.1136/bmjpo-2022-001571 (PMC9485650; doi:10.1136/bmjpo-2022-001571)
Supplement: Supplementary data [file bmjpo-2022-001571supp001.pdf]

SINEPOST
SARS-CoV-2 infection in neonates or in pregnancy: Outcomes at 18 months

Health Care Contacts

1. Has your child been admitted to hospital as an in-patient **since they were born**? If they were admitted to the neonatal unit soon after birth please **do not** count this admission.
   (*Please tick yes or no*)

   Yes ☐ No ☐

   If yes, please give the reason for **each** in-patient hospital admission and the number of nights spent in hospital in the table below

**In-patient admission**	**Reason for hospital in-patient admission**	**Number of nights spent in hospital**
1		
2		
3		
4		
5		
6		

If your child had more than six admissions, please provide details in the free text box on page 4.

2. Has your child been admitted to hospital as a day patient **since they were born**? This may have been for an operation or an investigation such as a scan where they **did not** need to spend the night in hospital. (*Please tick yes or no*)

   Yes ☐ No ☐

   If yes, please give the reason for **each** day patient hospital admission in the table below

**Day patient admission**	**Reason for hospital day patient admission**
1	
2	
3	
4	
5	
6	

Woodward K, *et al. BMJ Paediatrics Open* 2022; 6:e001571. doi: 10.1136/bmjpo-2022-001571

3. Has your child visited hospital for any of the following **since they were born**? (*please tick yes or no for all, and add total number of visits if yes)*

**Type of visit**	**Please tick**	**Total number of visits**
Outpatient clinic visit (eg. Paediatrician)	Yes ☐ No ☐	
Accident and Emergency department visit	Yes ☐ No ☐	
Other – please specify ______________________	Yes ☐ No ☐	

4. **Apart from** the routine health checks offered to all young children, has your child seen any of the following community professionals as a result of any other concerns **in the past 6 months?** (*Please indicate yes or no for all, and add total number of visits if yes*)

**Community Professional**	**Please tick**	**Total number of visits**
General Practitioner (GP)	Yes ☐ No ☐	
Health Visitor	Yes ☐ No ☐	
Practice Nurse	Yes ☐ No ☐	
Community Nurse	Yes ☐ No ☐	
Home visitor / volunteer	Yes ☐ No ☐	
Community children's doctor (Paediatrician)	Yes ☐ No ☐	
Physiotherapist	Yes ☐ No ☐	
Occupational Therapist	Yes ☐ No ☐	
Social Worker	Yes ☐ No ☐	
Optician	Yes ☐ No ☐	
Speech and Language therapist	Yes ☐ No ☐	
Dietitian	Yes ☐ No ☐	
Other – please specify ______________________	Yes ☐ No ☐	

Woodward K, *et al. BMJ Paediatrics Open* 2022; 6:e001571. doi: 10.1136/bmjpo-2022-001571

Personal Financial Costs

1. **Since your child was born** have you and/or partner had any additional personal financial costs **because of your child's health**? (*please tick all*)

**Examples of costs to you**	**Please tick**	**If yes, please give approximate total spent in £**	**Please give details**
Have you purchased special equipment?	Yes ☐ No ☐		
Have you made changes to your home because of your child's health?	Yes ☐ No ☐		
Have you paid excessively for travel and/or parking because of hospital or outpatient visits?	Yes ☐ No ☐		
Other – please specify	Yes ☐ No ☐		

2. **Since your child was born** have you and/or your partner had any time off work **as a result of your child's health**? (Please **do not** include time when you were on maternity/ paternity leave)

	**You**	**Your partner**
Have you and/or your partner taken time off work (**without pay**)?<br><br>*If Yes, how many days have you taken off work*	Yes ☐ No ☐<br><br>Days off work ______	Yes ☐ No ☐<br><br>Days off work ______
Have you and/or your partner taken time off work (**with pay**)?<br><br>*If Yes, how many days have you taken off work*	Yes ☐ No ☐<br><br>Days off work ______	Yes ☐ No ☐<br><br>Days off work ______

3. **Which of these best describes your current situation?** *(Please tick **only one** box in each column. If you are on maternity/paternity leave, please report what you expect your status to be when this leave ends)*

Woodward K, *et al. BMJ Paediatrics Open* 2022; 6:e001571. doi: 10.1136/bmjpo-2022-001571

**You**		**Your partner**	
In paid work full-time	☐	In paid work full-time	☐
In paid work part-time	☐	In paid work part-time	☐
At home looking after my family or dependents	☐	At home looking after my family or dependents	☐
In education	☐	In education	☐
Unemployed	☐	Unemployed	☐
Unable to work because of disability or ill health	☐	Unable to work because of disability or ill health	☐

If there were more than 6 hospital admissions, please provide the details in the box below.

**In-patient admission**	**Reason for hospital in-patient admission**	**Number of nights spent in the hospital**

Woodward K, *et al. BMJ Paediatrics Open* 2022; 6:e001571. doi: 10.1136/bmjpo-2022-001571
