# Supplementary material for: Effect of SARS-CoV-2 infection in neonates or in pregnancy on developmental outcomes at 21–24 months (SINEPOST): study protocol for a prospective cohort study
Source: BMJ Paediatr Open. 2022 Sep 19;6(1):e001571. doi: 10.1136/bmjpo-2022-001571 (PMC9485650; doi:10.1136/bmjpo-2022-001571)
Supplement: Author's manuscript [file bmjpo-2022-001571.draft_revisions.pdf]

BMJ Paediatrics Open

BMJ Paediatrics Open is committed to open peer review. As part of this commitment we make the peer review history of every article we publish publicly available.

When an article is published we post the peer reviewers' comments and the authors' responses online. We also post the versions of the paper that were used during peer review. These are the versions that the peer review comments apply to.

The versions of the paper that follow are the versions that were submitted during the peer review process. They are not the versions of record or the final published versions. They should not be cited or distributed as the published version of this manuscript.

BMJ Paediatrics Open is an open access journal and the full, final, typeset and author-corrected version of record of the manuscript is available on our site with no access controls, subscription charges or pay-per-view fees (http://bmjpaedsopen.bmj.com).

If you have any questions on BMJ Paediatrics Open's open peer review process please email info.bmjpo@bmj.com

BMJ Paediatrics Open

The effect of SARS-CoV-2 infection in neonates or in pregnancy on developmental outcomes at 21-24 months (SINEPOST): Study protocol for a prospective cohort study.

Journal:	*BMJ Paediatrics Open*
Manuscript ID	bmjpo-2022-001571
Article Type:	Protocol
Date Submitted by the Author:	07-Jun-2022
Complete List of Authors:	Woodward, Kathryn; University of Bristol, Translational Health Sciences, Bristol Medical School<br>Cornish, Rosie; University of Bristol, Population Health Sciences, Bristol Medical School<br>Gale, Chris; Imperial College London, Neonatal Medicine, School of Public Health, Faculty of Medicine<br>Johnson, Samantha; University of Leicester, Department of Health Sciences<br>Knight, Marian ; University of Oxford, NIHR Professor of Maternal and Child Population Health, NHIR Policy Research Unit in Maternal and Neonatal Health and Care, National Perinatal Epidemiology Unit, Nuffield Department of Population Health<br>Kurinczuk, Jenny; University of Oxford, Director National Perinatal Epidemiology Unit, NHIR Policy Research Unit in Maternal and Neonatal Health and Care, National Perinatal Epidemiology Unit, Nuffield Department of Population Health<br>Chakkarapani, Ela; University of Bristol, Translational Health Sciences, Bristol Medical School
Keywords:	COVID-19, Epidemiology, Neonatology, Psychology

SCHOLARONE™
Manuscripts

I, the Submitting Author has the right to grant and does grant on behalf of all authors of the Work (as defined in the below author licence), an exclusive licence and/or a non-exclusive licence for contributions from authors who are: i) UK Crown employees; ii) where BMJ has agreed a CC-BY licence shall apply, and/or iii) in accordance with the terms applicable for US Federal Government officers or employees acting as part of their official duties; on a worldwide, perpetual, irrevocable, royalty-free basis to BMJ Publishing Group Ltd ("BMJ") its licensees and where the relevant Journal is co-owned by BMJ to the co-owners of the Journal, to publish the Work in this journal and any other BMJ products and to exploit all rights, as set out in our licence.

The Submitting Author accepts and understands that any supply made under these terms is made by BMJ to the Submitting Author unless you are acting as an employee on behalf of your employer or a postgraduate student of an affiliated institution which is paying any applicable article publishing charge ("APC") for Open Access articles. Where the Submitting Author wishes to make the Work available on an Open Access basis (and intends to pay the relevant APC), the terms of reuse of such Open Access shall be governed by a Creative Commons licence – details of these licences and which Creative Commons licence will apply to this Work are set out in our licence referred to above.

Other than as permitted in any relevant BMJ Author's Self Archiving Policies, I confirm this Work has not been accepted for publication elsewhere, is not being considered for publication elsewhere and does not duplicate material already published. I confirm all authors consent to publication of this Work and authorise the granting of this licence.

Confidential: For Review Only

Title: The effect of SARS-CoV-2 infection in neonates or in pregnancy on developmental outcomes at 21-24 months (SINEPOST): Study protocol for a prospective cohort study.

Corresponding Author:

Ela Chakkarapani, FRCPCH MD, Translational Health Sciences, University of Bristol, Bristol BS2 8EG, UK

Tel: +44 (0) 117 342 5711

Fax: +44 (0) 117 34 5751

Email: ela.chakkarapani@bristol.ac.uk

Authors:

Kathryn Woodward, Translational Health Sciences, Bristol Medical School, University of Bristol, Bristol, UK

Rosie P Cornish, Population Health Sciences, Bristol Medical School, University of Bristol

Chris Gale, Neonatal Medicine, School of Public Health, Faculty of Medicine, Imperial College London, London, UK

Samantha Johnson, Department of Health Sciences, University of Leicester, Leicester, UK

Marian Knight, NIHR Professor of Maternal and Child Population Health, NIHR Policy Research Unit in Maternal and Neonatal Health and Care, National Perinatal Epidemiology Unit, Nuffield Department of Population Health, University of Oxford, Oxford, UK

Jennifer J Kurinczuk, Director National Perinatal Epidemiology Unit, NIHR Policy Research Unit in Maternal and Neonatal Health and Care, National Perinatal Epidemiology Unit, Nuffield Department of Population Health, University of Oxford, Oxford UK

Ela Chakkarapani, Translational Health Sciences, Bristol Medical School, University of Bristol, Bristol, UK

Word count: 2493

Confidential: For Review Only

Abstract

Introduction

Exposure to SARS-CoV-2 during pregnancy or in the neonatal period may impact fetal or neonatal brain development either through direct central nervous system infection or indirectly through the adverse effects of viral infection-related inflammation in the mother or newborn infant. This study aims to determine whether there are early neurodevelopmental effects of SARS-CoV-2 infection.

Methods and analysis

We will conduct a prospective national population-based cohort study of children aged 21-24 months who were born at term (≥37 weeks' gestation) between 1st March 2020 and 28th February 2021, and were either antenatally exposed, neonatally exposed, or unexposed (comparison cohort) to SARS-CoV-2. Nationally, hospitals will identify and approach parents of children eligible for inclusion in the antenatally and neonatally exposed cohorts using information from the UK Obstetric Surveillance System (UKOSS) and British Paediatric Surveillance Unit (BPSU) national surveillance studies and will identify and approach eligible children for the comparison cohort through routine birth records. Parents will be asked to complete questionnaires to assess their child's development at 21-24 months of age. Outcome measures comprise the Ages and Stages Questionnaire 3rd Edition (ASQ-3), Ages and Stages Questionnaire Social-Emotional 2nd edition (ASQ-SE-2), Liverpool respiratory symptoms questionnaire, and questionnaire items to elicit information about healthcare usage. With parental consent, study data will be linked to routine health and education records for future follow-up. Regression models will compare ASQ-3 and ASQ-SE-2 scores and proportions, frequency of respiratory symptoms, and healthcare usage between the exposed and comparison cohorts, adjusting for potential confounders.

Ethics and Dissemination

Ethics approval was obtained from the London-Westminster Research Ethics Committee. Findings will be disseminated in scientific conference presentations and peer-reviewed publications.

ISRCTN registry ID ISRCTN99910769

Confidential: For Review Only

Introduction

The SARS-CoV-2 virus has infected over 300 million people worldwide (1). Changes occurring to the immune system during pregnancy may predispose pregnant women to SARS-CoV-2 infection (2), and pregnant women with SARS-CoV-2 infection are at increased risk of requiring critical care (3,4). In a previous study, ninety-five percent of babies born to women with confirmed SARS-CoV-2 during pregnancy did not have a confirmed SARS-CoV-2 infection in the neonatal period (3). Where babies were infected in the neonatal period, possible transmission occurred from the mother in 26%, but this was most commonly in the postnatal period and vertical transmission appears to be rare (5). Overall the incidence of SARS-CoV-2 infection in neonates is low (5.6 per 10 000 live births) (5) and most neonates had mild symptoms or were asymptomatic (6). However, the long-term outcomes for these infants are unknown.

There are various possible mechanisms through which SARS-CoV-2 infection may adversely impact the developing fetus. SARS-CoV-2 infection triggers inflammation (7) which can pass through the placenta and may impact fetal brain development (8). Severe SARS-CoV-2 infection requiring critical care, which is more common in pregnancy, may impair uteroplacental blood flow inducing ischaemic brain injury to the fetus (9). SARS-CoV-2 may directly affect the fetal brain, as the placenta expresses the angiotensin converting enzyme 2 (ACE-2) receptor for SARS-CoV-2 (11), and SARS-CoV-2 has been detected in placental tissue (11–13) and amniotic fluid (14). Transplacental transmission has also been accompanied with neurological symptoms (13,15). However, based on the criteria for probable or possible intrapartum acquired neonatal SARS-CoV-2 infection (16), only 3% of hospitalised neonates were considered to have possible vertical transmission (5).

SARS-CoV-2 has been associated with increased incidence of neurological and cognitive deficits in adults (17,18). In adults with confirmed SARS-CoV-2 infection, SARS-CoV-2 RNA has been detected in brain tissue (19), and neuroimaging abnormalities have been observed in brain regions functionally-connected to the human olfactory system (20,21). Neuroimaging abnormalities have also been detected in white matter in an infant (15) and acute disseminated encephalomyelitis-like changes have been observed in the brains of children with acute and subacute SARS-CoV-2 infection (22). SARS-CoV-2 also evokes a widespread hyperinflammatory state with a 'cytokine storm' in some severely affected individuals (7), and severe disease is well described in pregnancy (3,4). In utero or early life exposure to high levels of inflammation (23,24) is known to be associated with cerebral white matter damage, leading to neurodevelopmental disorders in childhood and a range of long-term disabilities (25). Taken together, this suggests SARS-CoV-2 exposure in a fetus or neonate has the potential to adversely affect long-term neurodevelopment, either directly or indirectly via the effect of inflammation accompanying SARS-CoV-2 infection on the developing brain.

In the UK in March to April 2020, the majority of pregnant women who were hospitalised with SARS-CoV-2 were admitted in the late second or third trimester (3) and 73% gave birth at 37 weeks' gestation or later (5). Among neonatal cases of SARS-CoV-2 infection, two-thirds occurred in term-born babies (5). Enhanced developmental surveillance is recommended for preterm but not term-born infants; therefore, there is an urgent need to investigate the potential impact of antenatal or neonatal SARS-CoV-2 exposure on neurodevelopment in term-born infants. Given that SARS-CoV-2 infection impacts the respiratory system during acute infection in both neonates and adults (5,26), there may also be a long-term effect on lung function and consequently health care usage. Therefore, in addition to assessing neurodevelopment, we intend to establish whether there are any long-term impacts on respiratory function and health care usage.

Study aims and objectives

Confidential: For Review Only

This study aims to investigate neurodevelopmental effects of SARS-CoV-2 infection.

The primary objective of the study is to investigate the impact of antenatal and neonatal exposure to SARS-CoV-2 on infants' global development at 21-24 months of age.

The secondary objectives are to assess the impact of antenatal and neonatal SARS-CoV-2 exposure on:

- infants' cognitive, fine motor, gross motor, communication, and social-emotional development at 21-24 months of age.
- infants' respiratory symptoms at 21-24 months of age, and infant's utilisation of healthcare services.

Methods

Study design

A prospective national population-based cohort study of children aged 21-24 months with (exposed cohort) and without (comparison cohort) antenatal or neonatal exposure to SARS-CoV-2, who were born at term between 1st March 2020 and 28th February 2021.

The antenatal exposure cohort will include infants born to women with confirmed SARS-CoV-2 infection during pregnancy, and will be identified from the UK Obstetric Surveillance System (UKOSS) COVID-19 in pregnancy study (3). The neonatal exposure cohort will include infants with confirmed SARS-CoV-2 infection as a neonate, and will be identified from the British Paediatric Surveillance Unit (BPSU) Neonatal Complications of Coronavirus Disease study (5). These studies reported ongoing nationwide hospitalisation of pregnant women and neonates with SARS-CoV-2 infection, respectively. The comparison cohort will include infants born to women without evidence of SARS-CoV-2 infection during pregnancy, and whose baby did not have confirmed SARS-CoV-2 infection as a neonate, and these infants will be identified at the same hospitals that reported cases of maternal SARS-CoV-2 infection in pregnancy through the UKOSS study.

Inclusion criteria

- Antenatal exposure cohort includes term-born (≥37 weeks' gestation) singleton infants of mothers hospitalised with confirmed SARS-CoV-2 infection in pregnancy between 14 to 36 + 6 weeks gestation.
- Neonatal exposure cohort includes term-born singleton infants with confirmed SARS-CoV-2 infection who were hospitalised in the first 28 days after birth.
- Comparison cohort includes term-born singleton infants born during the same period as the exposed cohorts, and with no evidence of maternal SARS-CoV-2 infection in pregnancy or neonatal SARS-CoV-2 infection.

Exclusion criteria

- Children born preterm
- Children with a major congenital anomaly
- Children born following multiple pregnancy
- Children who have left the UK

Recruitment

The parents of eligible children will be approached and recruited through the hospitals that cared for the mother or child during SARS-CoV-2 infection or during birth. The study co-ordinator based at the

Confidential: For Review Only

National Perinatal Epidemiology Unit at the University of Oxford will send study information packs to the hospitals, where one study pack will be dispatched to the parent of each eligible child. The study information packs include:

1. Study invitation letter to parents of the eligible children
2. Parent information sheet
3. Form providing information in 7 non-English languages (Arabic, Gujarati, Hindi, Polish, Punjabi, Somali and Urdu) on how to find out more about the study in different languages.
4. Informed consent form
5. Non-participation form
6. Study entry questionnaire
7. Freepost return envelope

The local hospital research teams will be provided with the UKOSS ID for the mothers of antenatal exposure cohort participants and the BPSU ID, NHS number and date of birth of the neonatal exposure cohort participants. The hospital research teams will use these details to identify the names and addresses of the mothers and eligible children in the antenatal and neonatal exposure cohorts, respectively, from locally held information. The hospital research teams will also identify up to four babies who met the comparison cohort eligibility criteria and were born around the same time as each child with antenatal exposure using routine birth records – two babies born before and two babies born after the antenatal exposure baby, preferably on the same day. The hospital teams will also have the option to telephone the parents of eligible children and inform them about the study prior to posting each parent a study information pack.

Informed consent

Parents will be able to enrol their children onto the study by completing the consent form and study entry questionnaire and returning these forms to the central research team at the University of Bristol using the freepost return envelope. Parents who do not want to be involved in the study will be able to indicate this by returning the non-participation form. Parents will also be able to complete these forms online by following a weblink provided in the invitation letter. Parents who do not return any forms after three weeks will be sent one reminder study pack.

In addition to seeking parental consent for the child to take part in the study, the informed consent form will request consent to 1) retain the contact details of the parents to allow the research team to contact them to be invited to participate in future follow up assessments, 2) link the child's study data to records held by the Department for Education on academic attainment and special education needs (in the National Pupil Database), and 3) link the child's identifier to NHS Digital records (Hospital Episode Statistics and primary care attendance data).

Procedures and data collection

Data will be collected through parent-completed questionnaires, using paper copies returned by post to the central research team or online using a secure weblink. Parents who do not speak English will be able to complete the questionnaires over the phone with a researcher and translator.

Parents will complete the study entry questionnaire when providing informed written consent. This questionnaire will collect information about:

- Parents preferred choice of completing questionnaires (i.e., online, or via post or phone)
- The need to use translation services
- Demographic characteristics

Confidential: For Review Only

- SARS-CoV-2 infection status, and
- Information on other conditions that might impact neurodevelopment.

Parents will then be sent data collection materials when their child reaches 21-23 months of age. These will consist of the following questionnaires:

1) The Ages and Stages Questionnaire®, Third Edition (ASQ-3) 22-month Questionnaire (27), a widely used measure to assess children's development and identify children at risk of developmental delay (28). The ASQ-3 assesses development in 5 domains: fine motor, gross motor, communication, personal-social functioning and problem-solving.
2) The Ages and Stages Questionnaire®: Social-Emotional, Second Edition (ASQ-SE-2) 24-month Questionnaire (29), a widely used measure to identify children at risk of social-emotional problems (30).
3) Liverpool Respiratory Symptoms Questionnaire (LRSQ), developed to assess patterns of wheezing and other respiratory symptoms in infants and preschool children (31).
4) A non-validated questionnaire collecting information about the use of health care services and personal financial costs.

Parents who do not return the questionnaire within two weeks will be sent a reminder email or text message, and if there is no response after a month, parents will be contacted again. Parents who do not respond to invitations to complete the ASQ-3 before their child reaches 23 months old, will be sent the subsequent age band ASQ-3 24-month questionnaire. Parents will be informed of the results of their child's ASQ questionnaire results after completion. If the questionnaires highlight possible developmental delay according to standardised cut-offs, parents will be advised to contact their health visitor and/or general practitioner for further assessment.

Outcome measures

The primary outcome measure for this study is the mean total ASQ-3 score, and the secondary outcomes are:

1) Proportion with one or more ASQ-3 domain scores below the established cut-offs which identify possible developmental delay
2) Proportion of ASQ-3 domain scores below the established cut-offs which identify possible developmental delay
3) Mean ASQ-SE-2 total score & proportion of ASQ-SE-2 total scores above the established cut-offs which identify possible developmental delay
4) Mean LRSQ total score
5) Frequency of health care usage

Sample size

The study sample size aims to be able to detect a difference in neurodevelopment associated with perinatal SARS-CoV-2 exposure of similar magnitude to that seen in term-born infants with mild neonatal encephalopathy: a difference in developmental score of 6 points (0.5 SD) (32). Therefore, to detect a similarly clinically meaningful between-group difference of 0.5 SD in mean ASQ-3 total score (mean (SD) 203.02 (50.84)) (33) in exposed vs. comparison infants, with a Type-I error of 0.05 and 90% power, we will need a minimum of 85 infants in each of the two exposure groups and in the comparison group.

To achieve a sufficient sample size for future follow-up studies at school-age considering potential attrition, we aim to recruit at least 200 children in each group. Given the 20% response rate in a

Confidential: For Review Only

previous study of similar design using the UKOSS platform (34), we will approach the parents of up to 1000 children in the antenatal exposure cohort and up to 2000 children for the comparison cohort. Given that there are around 120 eligible children in the neonatal exposure cohort to date, we will approach the parents of all these infants. Consequently, our target sample size comprises 200 children in the antenatal exposure cohort, 200 in the comparison cohort and up to 120 children in the neonatal exposure cohort.

Data analysis

We will use descriptive characteristics to compare the exposed and comparison cohorts and assess their representativeness (against national surveillance data, and data from the Office for National Statistics and National Records for Scotland, for the exposed and comparison cohorts, respectively). We will use multiple linear and logistic regression to compare primary and secondary outcomes between the exposed and comparison cohorts, adjusting for potential confounders (e.g., baseline socio-demographic factors including ethnicity, sex, socio-economic status) and carry out exploratory sub-group analyses by gestation for antenatal exposure and severity of SARS-CoV-2 infection in the neonatal period. We will also conduct sensitivity analysis to evaluate the impact of children with other conditions that could impact neurodevelopment including hypoxic-ischaemic encephalopathy, seizures, perinatal stroke, bacterial or non-SARS-CoV-2 viral meningitis, and neuroimaging abnormalities in the neonatal period.

Discussion

The neuroinvasive potential of SARS-CoV-2 was speculated upon formulating the study protocol, as some adults with moderate/severe SARS-CoV-2 infection presented with neurological manifestations (35), raised neuronal injury and glial activation markers (36), and as the ACE-2 receptor for SARS-CoV-2 is expressed on neurons, glial cells and oligodendrocytes (37–39). This speculation has since been supported by evidence linking SARS-CoV-2 infection to brain abnormalities and cognitive deficits in adults (17,18,20,21).

Currently, there is a lack of representative studies examining the impact of maternal SARS-CoV-2 infection in pregnancy and neonatal SARS-CoV-2 infection on the long-term neurodevelopment outcomes of infants. Identifying whether children in these cohorts may be at increased risk of developmental delay is important, as this will inform public health advice provided by the NHS and clinicians to pregnant women and parents of newborn children exposed to SARS-CoV-2 infection.

Patient and public involvement

The study design was discussed with a patient and public involvement (PPI) group consisting of a parent of an infant exposed to SARS-CoV-2 in the antenatal period and another parent whose infant was exposed to SARS-CoV-2 in the neonatal period. The PPI group agreed that the research question is important and the design and outcomes to be measured are appropriate. The parent information sheet and consent form were developed with input from the PPI group, and further PPI will be sought to support the conduct of the study and help with interpreting the results.

Ethics and dissemination

Approval was obtained from London-Westminster Research Ethics Committee (REC; protocol number: 2021-93), and subsequent amendments to the protocol requiring REC approval were implemented after revised documents were reviewed and approved by the REC and participating organisations. Non-substantial amendments including the addition of new sites were reviewed by the sponsor,

Confidential: For Review Only

submitted to the HRA for information only, and shared with the participating organisations. Findings will be disseminated in scientific conference presentations and peer-reviewed publications.

Confidential: For Review Only

References

1. World Health Organization. WHO Coronavirus (COVID-19) Dashboard. WHO Coronavirus (COVID-19) Dashboard With Vaccination Data [Internet]. WHO. 2022 [cited 2022 Jan 25]. p. 1–5. Available from: https://covid19.who.int/

2. Rangchaikul P, Venketaraman V. SARS-CoV-2 and the Immune Response in Pregnancy with Delta Variant Considerations. Infect Dis Rep [Internet]. 2021 Nov 30 [cited 2022 Jan 25];13(4):993. Available from: /pmc/articles/PMC8700906/

3. Knight M, Bunch K, Vousden N, Morris E, Simpson N, Gale C, et al. Characteristics and outcomes of pregnant women admitted to hospital with confirmed SARS-CoV-2 infection in UK: national population based cohort study. BMJ [Internet]. 2020 Jun 8 [cited 2022 Jan 25];369. Available from: /pmc/articles/PMC7277610/

4. Allotey J, Stallings E, Bonet M, Yap M, Chatterjee S, Kew T, et al. Clinical manifestations, risk factors, and maternal and perinatal outcomes of coronavirus disease 2019 in pregnancy: living systematic review and meta-analysis. BMJ [Internet]. 2020 Sep 1 [cited 2022 Jan 17];370:3320. Available from: https://www.bmj.com/content/370/bmj.m3320

5. Gale C, Quigley MA, Placzek A, Knight M, Ladhani S, Draper ES, et al. Characteristics and outcomes of neonatal SARS-CoV-2 infection in the UK: a prospective national cohort study using active surveillance. Lancet Child Adolesc Heal. 2021 Feb 1;5(2):113–21.

6. Trevisanuto D, Cavallin F, Cavicchiolo ME, Borellini M, Calgaro S, Baraldi E. Coronavirus infection in neonates: a systematic review. Arch Dis Child - Fetal Neonatal Ed [Internet]. 2021 May 1 [cited 2022 Feb 2];106(3):330–5. Available from: https://fn.bmj.com/content/106/3/330

7. Tay MZ, Poh CM, Rénia L, MacAry PA, Ng LFP. The trinity of COVID-19: immunity, inflammation and intervention. Nat Rev Immunol [Internet]. 2020 Jun 1 [cited 2022 Jan 28];20(6):363–74. Available from: https://pubmed.ncbi.nlm.nih.gov/32346093/

8. Goeden N, Velasquez J, Arnold KA, Chan Y, Lund BT, Anderson GM, et al. Maternal Inflammation Disrupts Fetal Neurodevelopment via Increased Placental Output of Serotonin to the Fetal Brain. J Neurosci [Internet]. 2016 Jun 1 [cited 2022 Jan 28];36(22):6041. Available from: /pmc/articles/PMC4887568/

9. Di Girolamo R, Khalil A, Alameddine S, D'Angelo E, Galliani C, Matarrelli B, et al. Placental histopathology after SARS-CoV-2 infection in pregnancy: a systematic review and meta-analysis. Am J Obstet Gynecol Mfm [Internet]. 2021 Nov 1 [cited 2022 Jan 31];3(6):100468. Available from: /pmc/articles/PMC8379009/

10. Zheng QL, Duan T, Jin LP. Single-cell RNA expression profiling of ACE2 and AXL in the human maternal–Fetal interface. Reprod Dev Med [Internet]. 2020 Mar 25 [cited 2022 Jan 31];4(1):7. Available from: https://www.repdevmed.org/article.asp?issn=2096-2924;year=2020;volume=4;issue=1;spage=7;epage=10;aulast=Zheng

11. Taglauer E, Benarroch Y, Rop K, Barnett E, Sabharwal V, Yarrington C, et al. Consistent localization of SARS-CoV-2 spike glycoprotein and ACE2 over TMPRSS2 predominance in placental villi of 15 COVID-19 positive maternal-fetal dyads. Placenta [Internet]. 2020 Oct 1 [cited 2022 Jan 31];100:69–74. Available from: https://pubmed.ncbi.nlm.nih.gov/32862058/

12. Patanè L, Morotti D, Giunta MR, Sigismondi C, Piccoli MG, Frigerio L, et al. Vertical transmission of coronavirus disease 2019: severe acute respiratory syndrome coronavirus 2 RNA on the fetal side of the placenta in pregnancies with coronavirus disease 2019-positive

Confidential: For Review Only

mothers and neonates at birth. Am J Obstet Gynecol MFM [Internet]. 2020 Aug 1 [cited 2022 Jan 31];2(3). Available from: https://pubmed.ncbi.nlm.nih.gov/32427221/

13. Facchetti F, Bugatti M, Drera E, Tripodo C, Sartori E, Cancila V, et al. SARS-CoV2 vertical transmission with adverse effects on the newborn revealed through integrated immunohistochemical, electron microscopy and molecular analyses of Placenta. EBioMedicine. 2020 Sep 1;59:102951.

14. Zamaniyan M, Ebadi A, Aghajanpoor S, Rahmani Z, Haghshenas M, Azizi S. Preterm delivery, maternal death, and vertical transmission in a pregnant woman with COVID-19 infection. Prenat Diagn [Internet]. 2020 Dec 1 [cited 2022 Jan 31];40(13):1759–61. Available from: https://pubmed.ncbi.nlm.nih.gov/32304114/

15. Vivanti AJ, Vauloup-Fellous C, Prevot S, Zupan V, Suffee C, Do Cao J, et al. Transplacental transmission of SARS-CoV-2 infection. Nat Commun [Internet]. 2020 Dec 1 [cited 2022 Feb 1];11(1). Available from: https://pubmed.ncbi.nlm.nih.gov/32665677/

16. Shah PS, Diambomba Y, Acharya G, Morris SK, Bitnun A. Classification system and case definition for SARS-CoV-2 infection in pregnant women, fetuses, and neonates. Acta Obstet Gynecol Scand [Internet]. 2020 May 1 [cited 2022 Feb 1];99(5):565–8. Available from: https://pubmed.ncbi.nlm.nih.gov/32277845/

17. Taquet M, Luciano S, Geddes JR, Harrison PJ. Bidirectional associations between COVID-19 and psychiatric disorder: retrospective cohort studies of 62 354 COVID-19 cases in the USA. The Lancet Psychiatry [Internet]. 2021 Feb 1 [cited 2022 Mar 25];8(2):130–40. Available from: http://www.thelancet.com/article/S2215036620304624/fulltext

18. Liu Y-H, Chen Y, Wang Q-H, Wang L-R, Jiang L, Yang Y, et al. One-Year Trajectory of Cognitive Changes in Older Survivors of COVID-19 in Wuhan, China: A Longitudinal Cohort Study. JAMA Neurol [Internet]. 2022 Mar 8 [cited 2022 Mar 25]; Available from: https://pubmed.ncbi.nlm.nih.gov/35258587/

19. Chertow D, Stein S, Ramelli S, Grazioli A, Singh M, Yinda CK, et al. SARS-CoV-2 infection and persistence throughout the human body and brain. 2021 Dec 20 [cited 2022 Mar 25]; Available from: https://www.researchsquare.com

20. Douaud G, Lee S, Alfaro-Almagro F, Arthofer C, Wang C, Mccarthy P, et al. SARS-CoV-2 is associated with changes in brain structure in UK Biobank. [cited 2022 Mar 25]; Available from: https://doi.org/10.1038/s41586-022-04569-5

21. Politi LS, Salsano E, Grimaldi M. Magnetic Resonance Imaging Alteration of the Brain in a Patient With Coronavirus Disease 2019 (COVID-19) and Anosmia. JAMA Neurol [Internet]. 2020 Aug 1 [cited 2022 Feb 1];77(8):1028–9. Available from: https://jamanetwork.com/journals/jamaneurology/fullarticle/2766765

22. Lindan CE, Mankad K, Ram D, Kociolek LK, Silvera VM, Boddaert N, et al. Neuroimaging manifestations in children with SARS-CoV-2 infection: a multinational, multicentre collaborative study. Lancet Child Adolesc Heal [Internet]. 2021 Mar 1 [cited 2022 Feb 1];5(3):167–77. Available from: http://www.thelancet.com/article/S235246422030362X/fulltext

23. Brown AS, Meyer U. Maternal Immune Activation and Neuropsychiatric Illness: A Translational Research Perspective. Am J Psychiatry [Internet]. 2018 Nov 1 [cited 2022 Jan 28];175(11):1073–83. Available from: https://pubmed.ncbi.nlm.nih.gov/30220221/

24. Venkatesh KK, Leviton A, Hecht JL, Joseph RM, Douglass LM, Frazier JA, et al. Histologic

Confidential: For Review Only

chorioamnionitis and risk of neurodevelopmental impairment at age 10 years among extremely preterm infants born before 28 weeks of gestation. Am J Obstet Gynecol [Internet]. 2020 May 7 [cited 2022 Feb 1];223(5):745.e1-745.e10. Available from: https://europepmc.org/articles/PMC7609587

25. Leviton A, Dammann O, Durum SK. The adaptive immune response in neonatal cerebral white matter damage. Ann Neurol [Internet]. 2005 Dec [cited 2022 Feb 1];58(6):821–8. Available from: https://pubmed.ncbi.nlm.nih.gov/16250014/

26. Zhang H, Rostami MR, Leopold PL, Mezey JG, O'Beirne SL, Strulovici-Barel Y, et al. Expression of the SARS-CoV-2 ACE2 receptor in the human airway epithelium. Am J Respir Crit Care Med [Internet]. 2020 Jul 15 [cited 2022 Mar 28];202(2):219–29. Available from: www.ncbi.nlm.nih.gov/geo/

27. Squires, J., & Bricker D. Ages & Stages Questionnaires®, Third Edition (ASQ®-3): A Parent-Completed Child Monitoring System. Baltimore: Paul H. Brookes Publishing Co., Inc.; 2009.

28. Schonhaut L, Armijo I, Schönstedt M, Alvarez J, Cordero M. Validity of the ages and stages questionnaires in term and preterm infants. Pediatrics [Internet]. 2013 May [cited 2022 Jan 26];131(5). Available from: https://pubmed.ncbi.nlm.nih.gov/23629619/

29. Squires, J., Bricker, D., & Twombly E. Ages & Stages Questionnaires®: Social-Emotional, Second Edition (ASQ®:SE-2): A Parent-Completed Child Monitoring System for Social-Emotional Behaviors. Baltimore: Paul H. Brookes Publishing Co., Inc.; 2015.

30. Pontoppidan M, Nissa NK, Pejtersena JH, Julianc MM, Væverd MS. Parent report measures of infant and toddler social-emotional development: a systematic review. Fam Pract [Internet]. 2017 Apr 1 [cited 2022 Jan 26];34(2):127–37. Available from: https://academic.oup.com/fampra/article/34/2/127/2967465

31. Powell CVE, McNamara P, Solis A, Shaw NJ. A parent completed questionnaire to describe the patterns of wheezing and other respiratory symptoms in infants and preschool children. Arch Dis Child [Internet]. 2002 Nov 1 [cited 2022 Jan 26];87(5):376–9. Available from: https://pubmed.ncbi.nlm.nih.gov/12390904/

32. Finder M, Boylan GB, Twomey D, Ahearne C, Murray DM, Hallberg B. Two-Year Neurodevelopmental Outcomes After Mild Hypoxic Ischemic Encephalopathy in the Era of Therapeutic Hypothermia. JAMA Pediatr [Internet]. 2020 Jan 1 [cited 2022 Jan 26];174(1):48–55. Available from: https://pubmed.ncbi.nlm.nih.gov/31710357/

33. Steenis LJP, Verhoeven M, Hessen DJ, van Baar AL. Parental and professional assessment of early child development: the ASQ-3 and the Bayley-III-NL. Early Hum Dev [Internet]. 2015 Mar 1 [cited 2022 Jan 26];91(3):217–25. Available from: https://pubmed.ncbi.nlm.nih.gov/25703316/

34. Knight M, Acosta C, Brocklehurst P, Cheshire A, Fitzpatrick K, Hinton L, et al. Beyond maternal death: improving the quality of maternal care through national studies of 'near-miss' maternal morbidity. Program Grants Appl Res. 2016;4(9):1–180.

35. Varatharaj A, Thomas N, Ellul MA, Davies NWS, Pollak TA, Tenorio EL, et al. Neurological and neuropsychiatric complications of COVID-19 in 153 patients: a UK-wide surveillance study. The Lancet Psychiatry [Internet]. 2020 Oct 1 [cited 2022 Feb 1];7(10):875–82. Available from: http://www.thelancet.com/article/S221503662030287X/fulltext

36. Kanberg N, Ashton NJ, Andersson LM, Yilmaz A, Lindh M, Nilsson S, et al. Neurochemical evidence of astrocytic and neuronal injury commonly found in COVID-19. Neurology

Confidential: For Review Only

[Internet]. 2020 Sep 22 [cited 2022 Feb 1];95(12):e1754–9. Available from: https://n.neurology.org/content/95/12/e1754

37. Baig AM, Khaleeq A, Ali U, Syeda H. Evidence of the COVID-19 Virus Targeting the CNS: Tissue Distribution, Host-Virus Interaction, and Proposed Neurotropic Mechanisms. ACS Chem Neurosci [Internet]. 2020 Apr 1 [cited 2022 Feb 1];11(7):995–8. Available from: https://pubmed.ncbi.nlm.nih.gov/32167747/

38. Yamagata R, Nemoto W, Nakagawasai O, Takahashi K, Tan-No K. Downregulation of spinal angiotensin converting enzyme 2 is involved in neuropathic pain associated with type 2 diabetes mellitus in mice. Biochem Pharmacol [Internet]. 2020 Apr 1 [cited 2022 Feb 1];174. Available from: https://pubmed.ncbi.nlm.nih.gov/31987854/

39. Chen FZ, Wang Y, Sun XR, Yao YH, Zhang N, Qiao HF, et al. Emotional experiences predict the conversion of individuals with attenuated psychosis syndrome to psychosis: A 6-month follow up study. Front Psychol. 2016 Jun 1;7(JUN):818.

Confidential: For Review Only

Authors' contributions.

EC, CG, MK, JK, SJ and RC contributed to the conception and design of the study and KW drafted the manuscript, which was revised critically for important intellectual content by the other contributing authors. All authors approved the final version to be published. We also acknowledge the contributions from the co-collaborators on this study: K M Abel, H Mactier, S Doherty, S Ladhani, D Sharkey, E S Draper.

Funding statement

The research activities are funded by a project grant from Action Medical Research (GM2905). The BPSU study was funded by the National Institute for Health Research (NIHR) Policy Research Programme, conducted through the NIHR Policy Research Unit in Maternal and Neonatal Health and Care, PR-PRU-1217-21202. The views expressed are those of the author(s) and not necessarily those of the NIHR or the Department of Health and Social Care. The UKOSS study was funded by the NIHR Health Services and Delivery Research Programme (project number 11/46/12). MK is an NIHR senior investigator.

Competing interest's statement

There are no competing interests.

What is known about the subject?

- The incidence of vertical transmission of SARS-CoV-2, as well as the incidence of SARS-CoV-2 infection in neonates is low.
- Most neonates infected by SARS-CoV-2 had mild symptoms or were asymptomatic.
- SARS-CoV-2 has neuroinvasive potential and may impact children's neurodevelopment.

What this study hopes to add?

- A population-based evaluation of the impact of SARS-CoV-2 infection in pregnancy or in the neonatal period on children's neurodevelopment at 21-24 months of age.
- An assessment of healthcare usage and respiratory symptoms in children exposed to SARS-CoV-2 infection as a fetus or neonate compared to non-exposed children.

How this study might affect research, practice, or policy

- The outcomes of this study will inform public health advice provided by the NHS and clinicians to pregnant women and parents of newborn babies exposed to SARS-CoV-2 infection.

Confidential: For Review Only

BMJ Paediatrics Open

The effect of SARS-CoV-2 infection in neonates or in pregnancy on developmental outcomes at 21-24 months (SINEPOST): Study protocol for a prospective cohort study.

Journal:	*BMJ Paediatrics Open*
Manuscript ID	bmjpo-2022-001571.R1
Article Type:	Protocol
Date Submitted by the Author:	28-Jul-2022
Complete List of Authors:	Woodward, Kathryn; University of Bristol, Translational Health Sciences, Bristol Medical School<br>Cornish, Rosie; University of Bristol, Population Health Sciences, Bristol Medical School<br>Gale, Chris; Imperial College London, Neonatal Medicine, School of Public Health, Faculty of Medicine<br>Johnson, Samantha; University of Leicester, Department of Health Sciences<br>Knight, Marian ; University of Oxford, NIHR Professor of Maternal and Child Population Health, NHIR Policy Research Unit in Maternal and Neonatal Health and Care, National Perinatal Epidemiology Unit, Nuffield Department of Population Health<br>Kurinczuk, Jenny; University of Oxford, Director National Perinatal Epidemiology Unit, NHIR Policy Research Unit in Maternal and Neonatal Health and Care, National Perinatal Epidemiology Unit, Nuffield Department of Population Health<br>Chakkarapani, Ela; University of Bristol, Translational Health Sciences, Bristol Medical School
Keywords:	COVID-19, Epidemiology, Neonatology, Psychology

SCHOLARONE™
Manuscripts

I, the Submitting Author has the right to grant and does grant on behalf of all authors of the Work (as defined in the below author licence), an exclusive licence and/or a non-exclusive licence for contributions from authors who are: i) UK Crown employees; ii) where BMJ has agreed a CC-BY licence shall apply, and/or iii) in accordance with the terms applicable for US Federal Government officers or employees acting as part of their official duties; on a worldwide, perpetual, irrevocable, royalty-free basis to BMJ Publishing Group Ltd ("BMJ") its licensees and where the relevant Journal is co-owned by BMJ to the co-owners of the Journal, to publish the Work in this journal and any other BMJ products and to exploit all rights, as set out in our licence.

The Submitting Author accepts and understands that any supply made under these terms is made by BMJ to the Submitting Author unless you are acting as an employee on behalf of your employer or a postgraduate student of an affiliated institution which is paying any applicable article publishing charge ("APC") for Open Access articles. Where the Submitting Author wishes to make the Work available on an Open Access basis (and intends to pay the relevant APC), the terms of reuse of such Open Access shall be governed by a Creative Commons licence – details of these licences and which Creative Commons licence will apply to this Work are set out in our licence referred to above.

Other than as permitted in any relevant BMJ Author's Self Archiving Policies, I confirm this Work has not been accepted for publication elsewhere, is not being considered for publication elsewhere and does not duplicate material already published. I confirm all authors consent to publication of this Work and authorise the granting of this licence.

Confidential: For Review Only

Title: The effect of SARS-CoV-2 infection in neonates or in pregnancy on developmental outcomes at 21-24 months (SINEPOST): Study protocol for a prospective cohort study.

Corresponding Author:

Ela Chakkarapani, FRCPCH MD, Translational Health Sciences, University of Bristol, Bristol BS2 8EG, UK

Tel: +44 (0) 117 342 5711

Fax: +44 (0) 117 34 5751

Email: ela.chakkarapani@bristol.ac.uk

Authors:

Kathryn Woodward, Translational Health Sciences, Bristol Medical School, University of Bristol, Bristol, UK

Rosie P Cornish, Population Health Sciences, Bristol Medical School, University of Bristol

Chris Gale, Neonatal Medicine, School of Public Health, Faculty of Medicine, Imperial College London, London, UK

Samantha Johnson, Department of Health Sciences, University of Leicester, Leicester, UK

Marian Knight, NIHR Professor of Maternal and Child Population Health, NIHR Policy Research Unit in Maternal and Neonatal Health and Care, National Perinatal Epidemiology Unit, Nuffield Department of Population Health, University of Oxford, Oxford, UK

Jennifer J Kurinczuk, Director National Perinatal Epidemiology Unit, NIHR Policy Research Unit in Maternal and Neonatal Health and Care, National Perinatal Epidemiology Unit, Nuffield Department of Population Health, University of Oxford, Oxford UK

Ela Chakkarapani, Translational Health Sciences, Bristol Medical School, University of Bristol, Bristol, UK

Word count: 2493

Confidential: For Review Only

Abstract

Introduction

Exposure to SARS-CoV-2 during pregnancy or in the neonatal period may impact fetal or neonatal brain development either through direct central nervous system infection or indirectly through the adverse effects of viral infection-related inflammation in the mother or newborn infant. This study aims to determine whether there are early neurodevelopmental effects of SARS-CoV-2 infection.

Methods and analysis

We will conduct a prospective national population-based cohort study of children aged 21-24 months who were born at term (≥37 weeks' gestation) between 1st March 2020 and 28th February 2021, and were either antenatally exposed, neonatally exposed, or unexposed (comparison cohort) to SARS-CoV-2. Nationally, hospitals will identify and approach parents of children eligible for inclusion in the antenatally and neonatally exposed cohorts using information from the UK Obstetric Surveillance System (UKOSS) and British Paediatric Surveillance Unit (BPSU) national surveillance studies and will identify and approach eligible children for the comparison cohort through routine birth records. Parents will be asked to complete questionnaires to assess their child's development at 21-24 months of age. Outcome measures comprise the Ages and Stages Questionnaire 3rd Edition (ASQ-3), Ages and Stages Questionnaire Social-Emotional 2nd edition (ASQ-SE-2), Liverpool respiratory symptoms questionnaire, and questionnaire items to elicit information about healthcare usage. With parental consent, study data will be linked to routine health and education records for future follow-up. Regression models will compare ASQ-3 and ASQ-SE-2 scores and proportions, frequency of respiratory symptoms, and healthcare usage between the exposed and comparison cohorts, adjusting for potential confounders.

Ethics and Dissemination

Ethics approval was obtained from the London-Westminster Research Ethics Committee. Findings will be disseminated in scientific conference presentations and peer-reviewed publications.

ISRCTN registry ID ISRCTN99910769

Confidential: For Review Only

Introduction

The SARS-CoV-2 virus has infected over 300 million people worldwide (1). Changes occurring to the immune system during pregnancy may predispose pregnant women to SARS-CoV-2 infection (2), and pregnant women with SARS-CoV-2 infection are at increased risk of requiring critical care (3,4). In a previous study, ninety-five percent of babies born to women with confirmed SARS-CoV-2 during pregnancy did not have a confirmed SARS-CoV-2 infection in the neonatal period (3). Where babies were infected in the neonatal period, possible transmission occurred from the mother in 26%, but this was most commonly in the postnatal period and vertical transmission appears to be rare (5). Overall the incidence of SARS-CoV-2 infection in neonates is low (5.6 per 10 000 live births) (5) and most neonates had mild symptoms or were asymptomatic (6). However, the long-term outcomes for these infants are unknown.

There are various possible mechanisms through which SARS-CoV-2 infection may adversely impact the developing fetus. SARS-CoV-2 infection triggers inflammation (7) which can pass through the placenta and may impact fetal brain development (8). Severe SARS-CoV-2 infection requiring critical care, which is more common in pregnancy, may impair uteroplacental blood flow inducing ischaemic brain injury to the fetus (9). SARS-CoV-2 may directly affect the fetal brain, as the placenta expresses the angiotensin converting enzyme 2 (ACE-2) receptor for SARS-CoV-2 (11), and SARS-CoV-2 has been detected in placental tissue (11–13) and amniotic fluid (14). Transplacental transmission has also been accompanied with neurological symptoms (13,15). However, based on the criteria for probable or possible intrapartum acquired neonatal SARS-CoV-2 infection (16), only 3% of hospitalised neonates were considered to have possible vertical transmission (5).

SARS-CoV-2 has been associated with increased incidence of neurological and cognitive deficits in adults (17,18). In adults with confirmed SARS-CoV-2 infection, SARS-CoV-2 RNA has been detected in brain tissue (19), and neuroimaging abnormalities have been observed in brain regions functionally-connected to the human olfactory system (20,21). Neuroimaging abnormalities have also been detected in white matter in an infant (15) and acute disseminated encephalomyelitis-like changes have been observed in the brains of children with acute and subacute SARS-CoV-2 infection (22). SARS-CoV-2 also evokes a widespread hyperinflammatory state with a 'cytokine storm' in some severely affected individuals (7), and severe disease is well described in pregnancy (3,4). In utero or early life exposure to high levels of inflammation (23,24) is known to be associated with cerebral white matter damage, leading to neurodevelopmental disorders in childhood and a range of long-term disabilities (25). Taken together, this suggests SARS-CoV-2 exposure in a fetus or neonate has the potential to adversely affect long-term neurodevelopment, either directly or indirectly via the effect of inflammation accompanying SARS-CoV-2 infection on the developing brain.

In the UK in March to April 2020, the majority of pregnant women who were hospitalised with SARS-CoV-2 were admitted in the late second or third trimester (3) and 73% gave birth at 37 weeks' gestation or later (5). Among neonatal cases of SARS-CoV-2 infection, two-thirds occurred in term-born babies (5). Enhanced developmental surveillance is recommended for preterm but not term-born infants; therefore, there is an urgent need to investigate the potential impact of antenatal or neonatal SARS-CoV-2 exposure on neurodevelopment in term-born infants. Given that SARS-CoV-2 infection impacts the respiratory system during acute infection in both neonates and adults (5,26), there may also be a long-term effect on lung function and consequently health care usage. Therefore, in addition to assessing neurodevelopment, we intend to establish whether there are any long-term impacts on respiratory function and health care usage.

Study aims and objectives

https://mc.manuscriptcentral.com/bmjpo

Confidential: For Review Only

This study aims to investigate neurodevelopmental effects of SARS-CoV-2 infection.

The primary objective of the study is to investigate the impact of antenatal and neonatal exposure to SARS-CoV-2 on infants' global development at 21-24 months of age.

The secondary objectives are to assess the impact of antenatal and neonatal SARS-CoV-2 exposure on:

- infants' cognitive, fine motor, gross motor, communication, and social-emotional development at 21-24 months of age.
- infants' respiratory symptoms at 21-24 months of age, and infant's utilisation of healthcare services.

Methods

Study design

A prospective national population-based cohort study of children aged 21-24 months with (exposed cohort) and without (comparison cohort) antenatal or neonatal exposure to SARS-CoV-2, who were born at term between 1st March 2020 and 28th February 2021.

The antenatal exposure cohort will include infants born to women with confirmed SARS-CoV-2 infection during pregnancy, and will be identified from the UK Obstetric Surveillance System (UKOSS) COVID-19 in pregnancy study (3). The neonatal exposure cohort will include infants with confirmed SARS-CoV-2 infection as a neonate, and will be identified from the British Paediatric Surveillance Unit (BPSU) Neonatal Complications of Coronavirus Disease study (5). These studies reported ongoing nationwide hospitalisation of pregnant women and neonates with SARS-CoV-2 infection, respectively. The comparison cohort will include infants born to women without evidence of SARS-CoV-2 infection during pregnancy, and whose baby did not have confirmed SARS-CoV-2 infection as a neonate, and these infants will be identified at the same hospitals that reported cases of maternal SARS-CoV-2 infection in pregnancy through the UKOSS study.

Inclusion criteria

- Antenatal exposure cohort includes term-born (≥37 weeks' gestation) singleton infants of mothers hospitalised with confirmed SARS-CoV-2 infection in pregnancy between 14 to 36 + 6 weeks gestation.
- Neonatal exposure cohort includes term-born singleton infants with confirmed SARS-CoV-2 infection who were hospitalised in the first 28 days after birth.
- Comparison cohort includes term-born singleton infants born during the same period as the exposed cohorts, and with no evidence of maternal SARS-CoV-2 infection in pregnancy or neonatal SARS-CoV-2 infection.

Exclusion criteria

- Children born preterm
- Children with a major congenital anomaly
- Children born following multiple pregnancy
- Children who have left the UK

Recruitment

The parents of eligible children will be approached and recruited through the hospitals that cared for the mother or child during SARS-CoV-2 infection or during birth. The study co-ordinator based at the

Confidential: For Review Only

National Perinatal Epidemiology Unit at the University of Oxford will send study information packs to the hospitals, where one study pack will be dispatched to the parent of each eligible child. The study information packs include:

1. Study invitation letter to parents of the eligible children
2. Parent information sheet
3. Form providing information in 7 non-English languages (Arabic, Gujarati, Hindi, Polish, Punjabi, Somali and Urdu) on how to find out more about the study in different languages.
4. Informed consent form
5. Non-participation form
6. Study entry questionnaire
7. Freepost return envelope

The local hospital research teams will be provided with the UKOSS ID for the mothers of antenatal exposure cohort participants and the BPSU ID, NHS number and date of birth of the neonatal exposure cohort participants. The hospital research teams will use these details to identify the names and addresses of the mothers and eligible children in the antenatal and neonatal exposure cohorts, respectively, from locally held information. The hospital research teams will also identify up to four babies (depending on current recruitment rates) who met the comparison cohort eligibility criteria and were born around the same time as each child with antenatal exposure using routine birth records – two babies born before and two babies born after the antenatal exposure baby, preferably on the same day. The hospital teams will also have the option to telephone the parents of eligible children and inform them about the study prior to posting each parent a study information pack.

Informed consent

Parents will be able to enrol their children onto the study by completing the consent form and study entry questionnaire and returning these forms to the central research team at the University of Bristol using the freepost return envelope. Parents who do not want to be involved in the study will be able to indicate this by returning the non-participation form. Parents will also be able to complete these forms online by following a weblink provided in the invitation letter. Parents who do not return any forms after three weeks will be sent one reminder study pack.

In addition to seeking parental consent for the child to take part in the study, the informed consent form will request consent to 1) retain the contact details of the parents to allow the research team to contact them to be invited to participate in future follow up assessments, 2) link the child's study data to records held by the Department for Education on academic attainment and special education needs (in the National Pupil Database), and 3) link the child's identifier to NHS Digital records (Hospital Episode Statistics and primary care attendance data).

Procedures and data collection

Data will be collected through parent-completed questionnaires, either by parents returning paper copies to the central research team by post, or by completing the questionnaires online using a secure weblink or over the phone with the study researcher.

Parents will complete the study entry questionnaire when providing informed written consent. This questionnaire will collect information about:

- Parents preferred choice of completing questionnaires (i.e., online, or via post or phone)
- The need to use translation services
- Demographic characteristics

Confidential: For Review Only

- SARS-CoV-2 infection status, and
- Information on other conditions that might impact neurodevelopment.

Parents will then be sent data collection materials when their child reaches 21-23 months of age. These will consist of the following questionnaires:

1) The Ages and Stages Questionnaire®, Third Edition (ASQ-3) 22-month Questionnaire (27) in English; a widely used developmental screening tool to identify children at risk of developmental delay (28). The ASQ-3 assesses development in 5 domains: fine motor, gross motor, communication, personal-social functioning and problem-solving.
2) The Ages and Stages Questionnaire®: Social-Emotional, Second Edition (ASQ-SE-2) 24-month Questionnaire (29) in English; a widely used measure to identify children at risk of social-emotional problems (30).
3) Liverpool Respiratory Symptoms Questionnaire (LRSQ), developed to assess patterns of wheezing and other respiratory symptoms in infants and preschool children (31).
4) A non-validated questionnaire collecting information about the use of health care services and personal financial costs (Appendix 1).

We chose the ASQ-3 and ASQ-SE-2 as they are validated parent-completed questionnaires that are widely used to identify children at risk of development delay in multiple domains (28). Questionnaires will be completed over the telephone with the assistance of an interpreter for families who do not speak or read English sufficiently to complete the English version of the questionnaires. Parents who do not return the questionnaires within two weeks will be sent a reminder email or text message, and if there is no response after a month, parents will be contacted again. Parents who do not respond to invitations to complete the ASQ-3 before their child reaches 23 months old, will be sent the subsequent age band ASQ-3 24-month or 27-month questionnaire. Parents will be informed of the results of their child's ASQ questionnaire results after completion. If the questionnaires highlight possible developmental delay according to standardised cut-offs, parents will be advised to contact their health visitor and/or general practitioner for further assessment.

Outcome measures

The primary outcome measure for this study is the mean total ASQ-3 score, and the secondary outcomes are:

1) Proportion with one or more ASQ-3 domain scores below the established cut-offs which identify possible developmental delay
2) Proportion of ASQ-3 domain scores below the established cut-offs which identify possible developmental delay
3) Mean ASQ-SE-2 total score & proportion of ASQ-SE-2 total scores above the established cut-offs which identify possible developmental delay
4) Mean LRSQ total score
5) Frequency of health care usage

Sample size

The study sample size aims to be able to detect a difference in neurodevelopment associated with perinatal SARS-CoV-2 exposure of similar magnitude to that seen in term-born infants with mild neonatal encephalopathy: a difference in developmental score of 6 points (0.5 SD) (32). Therefore, to detect a similarly clinically meaningful between-group difference of 0.5 SD in mean ASQ-3 total score (mean (SD) 203.02 (50.84)) (33) in exposed vs. comparison infants, with a Type-I error of 0.05 and 90%

Confidential: For Review Only

power, we will need a minimum of 85 infants in each of the two exposure groups and in the comparison group.

To achieve a sufficient sample size for future follow-up studies at school-age considering potential attrition, we aim to recruit at least 200 children in each group. Given the 20% response rate in a previous study of similar design using the UKOSS platform (34), we will approach the parents of up to 1000 children in the antenatal exposure cohort (currently there are around 830 eligible antenatal exposure cohort children) and up to 2000 families for the comparison cohort, as we expect response rates to be lower for the comparison cohort. Given that there are around 120 eligible children in the neonatal exposure cohort to date, we will approach the parents of all these infants. Consequently, our target sample size comprises 200 children in the antenatal exposure cohort, 200 in the comparison cohort and up to 120 children in the neonatal exposure cohort.

Data analysis

We will use descriptive characteristics to compare the exposed and comparison cohorts and assess their representativeness (against national surveillance data, and data from the Office for National Statistics and National Records for Scotland, for the exposed and comparison cohorts, respectively). We will use multiple linear and logistic regression to compare primary and secondary outcomes between the exposed and comparison cohorts, adjusting for potential confounders (e.g., baseline socio-demographic factors including ethnicity, sex, socio-economic status) and carry out exploratory sub-group analyses by gestation for antenatal exposure and severity of SARS-CoV-2 infection in the neonatal period. We will also conduct sensitivity analysis to evaluate the impact of children with other conditions that could impact neurodevelopment including hypoxic-ischaemic encephalopathy, seizures, perinatal stroke, bacterial or non-SARS-CoV-2 viral meningitis, and neuroimaging abnormalities in the neonatal period.

Discussion

The neuroinvasive potential of SARS-CoV-2 was speculated upon formulating the study protocol, as some adults with moderate/severe SARS-CoV-2 infection presented with neurological manifestations (35), raised neuronal injury and glial activation markers (36), and as the ACE-2 receptor for SARS-CoV-2 is expressed on neurons, glial cells and oligodendrocytes (37–39). This speculation has since been supported by evidence linking SARS-CoV-2 infection to brain abnormalities and cognitive deficits in adults (17,18,20,21).

Currently, there is a lack of representative studies examining the impact of maternal SARS-CoV-2 infection in pregnancy and neonatal SARS-CoV-2 infection on the long-term neurodevelopment outcomes of infants. Identifying whether children in these cohorts may be at increased risk of developmental delay is important, as this will inform public health advice provided by the NHS and clinicians to pregnant women and parents of newborn children exposed to SARS-CoV-2 infection.

Patient and public involvement

The study design was discussed with a patient and public involvement (PPI) group consisting of a parent of an infant exposed to SARS-CoV-2 in the antenatal period and another parent whose infant was exposed to SARS-CoV-2 in the neonatal period. The PPI group agreed that the research question is important and the design and outcomes to be measured are appropriate. The parent information sheet and consent form were developed with input from the PPI group, and further PPI will be sought to support the conduct of the study and help with interpreting the results.

Ethics and dissemination

Confidential: For Review Only

Approval was obtained from London-Westminster Research Ethics Committee (REC; protocol number: 2021-93), and subsequent amendments to the protocol requiring REC approval were implemented after revised documents were reviewed and approved by the REC and participating organisations. Non-substantial amendments including the addition of new sites were reviewed by the sponsor, submitted to the HRA for information only, and shared with the participating organisations. Findings will be disseminated in scientific conference presentations and peer-reviewed publications.

Confidential: For Review Only

References

1. World Health Organization. WHO Coronavirus (COVID-19) Dashboard. WHO Coronavirus (COVID-19) Dashboard With Vaccination Data [Internet]. WHO. 2022 [cited 2022 Jan 25]. p. 1–5. Available from: https://covid19.who.int/

2. Rangchaikul P, Venketaraman V. SARS-CoV-2 and the Immune Response in Pregnancy with Delta Variant Considerations. Infect Dis Rep [Internet]. 2021 Nov 30 [cited 2022 Jan 25];13(4):993. Available from: /pmc/articles/PMC8700906/

3. Knight M, Bunch K, Vousden N, Morris E, Simpson N, Gale C, et al. Characteristics and outcomes of pregnant women admitted to hospital with confirmed SARS-CoV-2 infection in UK: national population based cohort study. BMJ [Internet]. 2020 Jun 8 [cited 2022 Jan 25];369. Available from: /pmc/articles/PMC7277610/

4. Allotey J, Stallings E, Bonet M, Yap M, Chatterjee S, Kew T, et al. Clinical manifestations, risk factors, and maternal and perinatal outcomes of coronavirus disease 2019 in pregnancy: living systematic review and meta-analysis. BMJ [Internet]. 2020 Sep 1 [cited 2022 Jan 17];370:3320. Available from: https://www.bmj.com/content/370/bmj.m3320

5. Gale C, Quigley MA, Placzek A, Knight M, Ladhani S, Draper ES, et al. Characteristics and outcomes of neonatal SARS-CoV-2 infection in the UK: a prospective national cohort study using active surveillance. Lancet Child Adolesc Heal. 2021 Feb 1;5(2):113–21.

6. Trevisanuto D, Cavallin F, Cavicchiolo ME, Borellini M, Calgaro S, Baraldi E. Coronavirus infection in neonates: a systematic review. Arch Dis Child - Fetal Neonatal Ed [Internet]. 2021 May 1 [cited 2022 Feb 2];106(3):330–5. Available from: https://fn.bmj.com/content/106/3/330

7. Tay MZ, Poh CM, Rénia L, MacAry PA, Ng LFP. The trinity of COVID-19: immunity, inflammation and intervention. Nat Rev Immunol [Internet]. 2020 Jun 1 [cited 2022 Jan 28];20(6):363–74. Available from: https://pubmed.ncbi.nlm.nih.gov/32346093/

8. Goeden N, Velasquez J, Arnold KA, Chan Y, Lund BT, Anderson GM, et al. Maternal Inflammation Disrupts Fetal Neurodevelopment via Increased Placental Output of Serotonin to the Fetal Brain. J Neurosci [Internet]. 2016 Jun 1 [cited 2022 Jan 28];36(22):6041. Available from: /pmc/articles/PMC4887568/

9. Di Girolamo R, Khalil A, Alameddine S, D'Angelo E, Galliani C, Matarrelli B, et al. Placental histopathology after SARS-CoV-2 infection in pregnancy: a systematic review and meta-analysis. Am J Obstet Gynecol Mfm [Internet]. 2021 Nov 1 [cited 2022 Jan 31];3(6):100468. Available from: /pmc/articles/PMC8379009/

10. Zheng QL, Duan T, Jin LP. Single-cell RNA expression profiling of ACE2 and AXL in the human maternal–Fetal interface. Reprod Dev Med [Internet]. 2020 Mar 25 [cited 2022 Jan 31];4(1):7. Available from: https://www.repdevmed.org/article.asp?issn=2096-2924;year=2020;volume=4;issue=1;spage=7;epage=10;aulast=Zheng

11. Taglauer E, Benarroch Y, Rop K, Barnett E, Sabharwal V, Yarrington C, et al. Consistent localization of SARS-CoV-2 spike glycoprotein and ACE2 over TMPRSS2 predominance in placental villi of 15 COVID-19 positive maternal-fetal dyads. Placenta [Internet]. 2020 Oct 1 [cited 2022 Jan 31];100:69–74. Available from: https://pubmed.ncbi.nlm.nih.gov/32862058/

12. Patanè L, Morotti D, Giunta MR, Sigismondi C, Piccoli MG, Frigerio L, et al. Vertical transmission of coronavirus disease 2019: severe acute respiratory syndrome coronavirus 2 RNA on the fetal side of the placenta in pregnancies with coronavirus disease 2019-positive

Confidential: For Review Only

mothers and neonates at birth. Am J Obstet Gynecol MFM [Internet]. 2020 Aug 1 [cited 2022 Jan 31];2(3). Available from: https://pubmed.ncbi.nlm.nih.gov/32427221/

13. Facchetti F, Bugatti M, Drera E, Tripodo C, Sartori E, Cancila V, et al. SARS-CoV2 vertical transmission with adverse effects on the newborn revealed through integrated immunohistochemical, electron microscopy and molecular analyses of Placenta. EBioMedicine. 2020 Sep 1;59:102951.

14. Zamaniyan M, Ebadi A, Aghajanpoor S, Rahmani Z, Haghshenas M, Azizi S. Preterm delivery, maternal death, and vertical transmission in a pregnant woman with COVID-19 infection. Prenat Diagn [Internet]. 2020 Dec 1 [cited 2022 Jan 31];40(13):1759–61. Available from: https://pubmed.ncbi.nlm.nih.gov/32304114/

15. Vivanti AJ, Vauloup-Fellous C, Prevot S, Zupan V, Suffee C, Do Cao J, et al. Transplacental transmission of SARS-CoV-2 infection. Nat Commun [Internet]. 2020 Dec 1 [cited 2022 Feb 1];11(1). Available from: https://pubmed.ncbi.nlm.nih.gov/32665677/

16. Shah PS, Diambomba Y, Acharya G, Morris SK, Bitnun A. Classification system and case definition for SARS-CoV-2 infection in pregnant women, fetuses, and neonates. Acta Obstet Gynecol Scand [Internet]. 2020 May 1 [cited 2022 Feb 1];99(5):565–8. Available from: https://pubmed.ncbi.nlm.nih.gov/32277845/

17. Taquet M, Luciano S, Geddes JR, Harrison PJ. Bidirectional associations between COVID-19 and psychiatric disorder: retrospective cohort studies of 62 354 COVID-19 cases in the USA. The Lancet Psychiatry [Internet]. 2021 Feb 1 [cited 2022 Mar 25];8(2):130–40. Available from: http://www.thelancet.com/article/S2215036620304624/fulltext

18. Liu Y-H, Chen Y, Wang Q-H, Wang L-R, Jiang L, Yang Y, et al. One-Year Trajectory of Cognitive Changes in Older Survivors of COVID-19 in Wuhan, China: A Longitudinal Cohort Study. JAMA Neurol [Internet]. 2022 Mar 8 [cited 2022 Mar 25]; Available from: https://pubmed.ncbi.nlm.nih.gov/35258587/

19. Chertow D, Stein S, Ramelli S, Grazioli A, Singh M, Yinda CK, et al. SARS-CoV-2 infection and persistence throughout the human body and brain. 2021 Dec 20 [cited 2022 Mar 25]; Available from: https://www.researchsquare.com

20. Douaud G, Lee S, Alfaro-Almagro F, Arthofer C, Wang C, Mccarthy P, et al. SARS-CoV-2 is associated with changes in brain structure in UK Biobank. [cited 2022 Mar 25]; Available from: https://doi.org/10.1038/s41586-022-04569-5

21. Politi LS, Salsano E, Grimaldi M. Magnetic Resonance Imaging Alteration of the Brain in a Patient With Coronavirus Disease 2019 (COVID-19) and Anosmia. JAMA Neurol [Internet]. 2020 Aug 1 [cited 2022 Feb 1];77(8):1028–9. Available from: https://jamanetwork.com/journals/jamaneurology/fullarticle/2766765

22. Lindan CE, Mankad K, Ram D, Kociolek LK, Silvera VM, Boddaert N, et al. Neuroimaging manifestations in children with SARS-CoV-2 infection: a multinational, multicentre collaborative study. Lancet Child Adolesc Heal [Internet]. 2021 Mar 1 [cited 2022 Feb 1];5(3):167–77. Available from: http://www.thelancet.com/article/S235246422030362X/fulltext

23. Brown AS, Meyer U. Maternal Immune Activation and Neuropsychiatric Illness: A Translational Research Perspective. Am J Psychiatry [Internet]. 2018 Nov 1 [cited 2022 Jan 28];175(11):1073–83. Available from: https://pubmed.ncbi.nlm.nih.gov/30220221/

24. Venkatesh KK, Leviton A, Hecht JL, Joseph RM, Douglass LM, Frazier JA, et al. Histologic

Confidential: For Review Only

chorioamnionitis and risk of neurodevelopmental impairment at age 10 years among extremely preterm infants born before 28 weeks of gestation. Am J Obstet Gynecol [Internet]. 2020 May 7 [cited 2022 Feb 1];223(5):745.e1-745.e10. Available from: https://europepmc.org/articles/PMC7609587

25. Leviton A, Dammann O, Durum SK. The adaptive immune response in neonatal cerebral white matter damage. Ann Neurol [Internet]. 2005 Dec [cited 2022 Feb 1];58(6):821–8. Available from: https://pubmed.ncbi.nlm.nih.gov/16250014/

26. Zhang H, Rostami MR, Leopold PL, Mezey JG, O'Beirne SL, Strulovici-Barel Y, et al. Expression of the SARS-CoV-2 ACE2 receptor in the human airway epithelium. Am J Respir Crit Care Med [Internet]. 2020 Jul 15 [cited 2022 Mar 28];202(2):219–29. Available from: www.ncbi.nlm.nih.gov/geo/

27. Squires, J., & Bricker D. Ages & Stages Questionnaires®, Third Edition (ASQ®-3): A Parent-Completed Child Monitoring System. Baltimore: Paul H. Brookes Publishing Co., Inc.; 2009.

28. Schonhaut L, Armijo I, Schönstedt M, Alvarez J, Cordero M. Validity of the ages and stages questionnaires in term and preterm infants. Pediatrics [Internet]. 2013 May [cited 2022 Jan 26];131(5). Available from: https://pubmed.ncbi.nlm.nih.gov/23629619/

29. Squires, J., Bricker, D., & Twombly E. Ages & Stages Questionnaires®: Social-Emotional, Second Edition (ASQ®:SE-2): A Parent-Completed Child Monitoring System for Social-Emotional Behaviors. Baltimore: Paul H. Brookes Publishing Co., Inc.; 2015.

30. Pontoppidan M, Nissa NK, Pejtersena JH, Julianc MM, Væverd MS. Parent report measures of infant and toddler social-emotional development: a systematic review. Fam Pract [Internet]. 2017 Apr 1 [cited 2022 Jan 26];34(2):127–37. Available from: https://academic.oup.com/fampra/article/34/2/127/2967465

31. Powell CVE, McNamara P, Solis A, Shaw NJ. A parent completed questionnaire to describe the patterns of wheezing and other respiratory symptoms in infants and preschool children. Arch Dis Child [Internet]. 2002 Nov 1 [cited 2022 Jan 26];87(5):376–9. Available from: https://pubmed.ncbi.nlm.nih.gov/12390904/

32. Finder M, Boylan GB, Twomey D, Ahearne C, Murray DM, Hallberg B. Two-Year Neurodevelopmental Outcomes After Mild Hypoxic Ischemic Encephalopathy in the Era of Therapeutic Hypothermia. JAMA Pediatr [Internet]. 2020 Jan 1 [cited 2022 Jan 26];174(1):48–55. Available from: https://pubmed.ncbi.nlm.nih.gov/31710357/

33. Steenis LJP, Verhoeven M, Hessen DJ, van Baar AL. Parental and professional assessment of early child development: the ASQ-3 and the Bayley-III-NL. Early Hum Dev [Internet]. 2015 Mar 1 [cited 2022 Jan 26];91(3):217–25. Available from: https://pubmed.ncbi.nlm.nih.gov/25703316/

34. Knight M, Acosta C, Brocklehurst P, Cheshire A, Fitzpatrick K, Hinton L, et al. Beyond maternal death: improving the quality of maternal care through national studies of 'near-miss' maternal morbidity. Program Grants Appl Res. 2016;4(9):1–180.

35. Varatharaj A, Thomas N, Ellul MA, Davies NWS, Pollak TA, Tenorio EL, et al. Neurological and neuropsychiatric complications of COVID-19 in 153 patients: a UK-wide surveillance study. The Lancet Psychiatry [Internet]. 2020 Oct 1 [cited 2022 Feb 1];7(10):875–82. Available from: http://www.thelancet.com/article/S221503662030287X/fulltext

36. Kanberg N, Ashton NJ, Andersson LM, Yilmaz A, Lindh M, Nilsson S, et al. Neurochemical evidence of astrocytic and neuronal injury commonly found in COVID-19. Neurology

Confidential: For Review Only

[Internet]. 2020 Sep 22 [cited 2022 Feb 1];95(12):e1754–9. Available from: https://n.neurology.org/content/95/12/e1754

37. Baig AM, Khaleeq A, Ali U, Syeda H. Evidence of the COVID-19 Virus Targeting the CNS: Tissue Distribution, Host-Virus Interaction, and Proposed Neurotropic Mechanisms. ACS Chem Neurosci [Internet]. 2020 Apr 1 [cited 2022 Feb 1];11(7):995–8. Available from: https://pubmed.ncbi.nlm.nih.gov/32167747/

38. Yamagata R, Nemoto W, Nakagawasai O, Takahashi K, Tan-No K. Downregulation of spinal angiotensin converting enzyme 2 is involved in neuropathic pain associated with type 2 diabetes mellitus in mice. Biochem Pharmacol [Internet]. 2020 Apr 1 [cited 2022 Feb 1];174. Available from: https://pubmed.ncbi.nlm.nih.gov/31987854/

39. Chen FZ, Wang Y, Sun XR, Yao YH, Zhang N, Qiao HF, et al. Emotional experiences predict the conversion of individuals with attenuated psychosis syndrome to psychosis: A 6-month follow up study. Front Psychol. 2016 Jun 1;7(JUN):818.

Confidential: For Review Only

Authors' contributions.

EC, CG, MK, JK, SJ and RC contributed to the conception and design of the study and KW drafted the manuscript, which was revised critically for important intellectual content by the other contributing authors. All authors approved the final version to be published. We also acknowledge the contributions from the co-collaborators on this study: K M Abel, H Mactier, S Doherty, S Ladhani, D Sharkey, E S Draper.

Funding statement

The research activities are funded by a project grant from Action Medical Research (GM2905). The BPSU study was funded by the National Institute for Health Research (NIHR) Policy Research Programme, conducted through the NIHR Policy Research Unit in Maternal and Neonatal Health and Care, PR-PRU-1217-21202. The views expressed are those of the author(s) and not necessarily those of the NIHR or the Department of Health and Social Care. The UKOSS study was funded by the NIHR Health Services and Delivery Research Programme (project number 11/46/12). MK is an NIHR senior investigator.

Competing interest's statement

There are no competing interests.

What is known about the subject?

- The incidence of vertical transmission of SARS-CoV-2, as well as the incidence of SARS-CoV-2 infection in neonates is low.
- Most neonates infected by SARS-CoV-2 had mild symptoms or were asymptomatic.
- SARS-CoV-2 has neuroinvasive potential and may impact children's neurodevelopment.

What this study hopes to add?

- A population-based evaluation of the impact of SARS-CoV-2 infection in pregnancy or in the neonatal period on children's neurodevelopment at 21-24 months of age.
- An assessment of healthcare usage and respiratory symptoms in children exposed to SARS-CoV-2 infection as a fetus or neonate compared to non-exposed children.

How this study might affect research, practice, or policy

- The outcomes of this study will inform public health advice provided by the NHS and clinicians to pregnant women and parents of newborn babies exposed to SARS-CoV-2 infection.

Confidential: For Review Only

SINEPOST
SARS-CoV-2 infection in neonates or in pregnancy: Outcomes at 18 months

Health Care Contacts

1. Has your child been admitted to hospital as an in-patient **since they were born**? If they were admitted to the neonatal unit soon after birth please **do not** count this admission. (*Please tick yes or no*)

Yes ☐ No ☐

If yes, please give the reason for **each** in-patient hospital admission and the number of nights spent in hospital in the table below

In-patient admission	Reason for hospital in-patient admission	Number of nights spent in hospital
1		
2		
3		
4		
5		
6		

If your child had more than six admissions, please provide details in the free text box on page 4.

2. Has your child been admitted to hospital as a day patient **since they were born**? This may have been for an operation or an investigation such as a scan where they **did not** need to spend the night in hospital. (*Please tick yes or no*)

Yes ☐ No ☐

If yes, please give the reason for **each** day patient hospital admission in the table below

Day patient admission	Reason for hospital day patient admission
1	
2	
3	
4	
5	
6	

Confidential: For Review Only

3. Has your child visited hospital for any of the following **since they were born**? (*please tick yes or no for all, and add total number of visits if yes)*

**Type of visit**	**Please tick**	**Total number of visits**
Outpatient clinic visit (eg. Paediatrician)	Yes ☐ No ☐	
Accident and Emergency department visit	Yes ☐ No ☐	
Other – please specify ____________________	Yes ☐ No ☐	

4. **Apart from** the routine health checks offered to all young children, has your child seen any of the following community professionals as a result of any other concerns **in the past 6 months?** (*Please indicate yes or no for all, and add total number of visits if yes*)

**Community Professional**	**Please tick**	**Total number of visits**
General Practitioner (GP)	Yes ☐ No ☐	
Health Visitor	Yes ☐ No ☐	
Practice Nurse	Yes ☐ No ☐	
Community Nurse	Yes ☐ No ☐	
Home visitor / volunteer	Yes ☐ No ☐	
Community children's doctor (Paediatrician)	Yes ☐ No ☐	
Physiotherapist	Yes ☐ No ☐	
Occupational Therapist	Yes ☐ No ☐	
Social Worker	Yes ☐ No ☐	
Optician	Yes ☐ No ☐	
Speech and Language therapist	Yes ☐ No ☐	
Dietitian	Yes ☐ No ☐	
Other – please specify ____________________	Yes ☐ No ☐	

Confidential: For Review Only

Personal Financial Costs

1. **Since your child was born** have you and/or partner had any additional personal financial costs **because of your child's health**? (*please tick all*)

**Examples of costs to you**	**Please tick**	**If yes, please give approximate total spent in £**	**Please give details**
Have you purchased special equipment?	Yes ☐ No ☐		
Have you made changes to your home because of your child's health?	Yes ☐ No ☐		
Have you paid excessively for travel and/or parking because of hospital or outpatient visits?	Yes ☐ No ☐		
Other – please specify	Yes ☐ No ☐		

2. **Since your child was born** have you and/or your partner had any time off work **as a result of your child's health**? (Please **do not** include time when you were on maternity/ paternity leave)

	**You**	**Your partner**
Have you and/or your partner taken time off work (**without pay**)?<br><br>*If Yes, how many days have you taken off work*	Yes ☐ No ☐<br><br>Days off work ______	Yes ☐ No ☐<br><br>Days off work ______
Have you and/or your partner taken time off work (**with pay**)?<br><br>*If Yes, how many days have you taken off work*	Yes ☐ No ☐<br><br>Days off work ______	Yes ☐ No ☐<br><br>Days off work ______

Confidential: For Review Only
https://mc.manuscriptcentral.com/bmjpo

3. **Which of these best describes your current situation?** *(Please tick* ***only one*** *box in each column. If you are on maternity/paternity leave, please report what you expect your status to be when this leave ends)*

**You**		**Your partner**	
In paid work full-time	☐	In paid work full-time	☐
In paid work part-time	☐	In paid work part-time	☐
At home looking after my family or dependents	☐	At home looking after my family or dependents	☐
In education	☐	In education	☐
Unemployed	☐	Unemployed	☐
Unable to work because of disability or ill health	☐	Unable to work because of disability or ill health	☐

If there were more than 6 hospital admissions, please provide the details in the box below.

**In-patient admission**	**Reason for hospital in-patient admission**	**Number of nights spent in the hospital**

Confidential: For Review Only
